# Vulnerability in the Context of Migration: a Critical Overview and a New Conceptual Model

**DOI:** 10.1007/s42087-022-00288-5

**Published:** 2022-04-19

**Authors:** Amalia Gilodi, Isabelle Albert, Birte Nienaber

**Affiliations:** 1 Department of Behavioural and Cognitive Sciences and Department of Geography and Spatial Planning, University of Luxembourg, Esch-sur-Alzette, Luxembourg; 2 Department of Behavioural and Cognitive Sciences, University of Luxembourg, Esch-sur-Alzette, Luxembourg; 3 Department of Geography and Spatial Planning, University of Luxembourg, Esch-sur-Alzette, Luxembourg

**Keywords:** Vulnerability, Migration, Refugees, Conceptual model, Migration policy

## Abstract

The notion of “vulnerability” occupies a central role in academic literature, policymaking, humanitarian debates, and everyday discourses on migration and asylum. Its popularity has led some academics and practitioners to use “vulnerability” as a self-explanatory condition or phenomenon. However, a common and systematic understanding of the concept is still missing, and the moral and political meaning often ascribed to this notion may have (un)intended detrimental consequences for those migrants deemed vulnerable. Thus, this paper sets out to critically unpack and highlight the complexities hidden behind this notion in order to provide a conceptual analysis of vulnerability in the context of migration. We do so by (1) providing an overview of definitions of vulnerability across different fields of research, (2) identifying common conceptualizations or types of vulnerability and discussing their implications, and (3) highlighting possible negative societal and psychological consequences of its implementation in the context of migration. Finally, we propose (4) a new conceptual model for understanding vulnerability in the context of migration, showing how this notion can become a useful analytical tool in migration research.

## Introduction

The terms “vulnerability” and “vulnerable groups” have become increasingly prominent in academic literature, policymaking, political debates, and everyday discourses on migration and asylum (Flegar, 2018; Heidbrink, 2020; Hruschka & Leboeuf, 2019; Monno & Serreli, 2020; Ní Raghallaigh & Thornton, 2017; Sözer, 2020). These terms have been widely employed in international humanitarian aid frameworks and legal documents by the most prominent international organizations in the field, such as the UNHCR and IOM, as well as in the New York Declaration for Refugees and Migrants adopted by the UN in 2016 (Flegar, 2018; United Nations, 2016). Special attention is paid to “vulnerable persons” also in the context of the Common European Asylum System (CEAS), such as in the recast Qualification Directive (2011/95/EU),^1^ the recast Asylum Procedures Directive (Directive 2013/32/EU),^2^ and particularly in the recast Reception Conditions Directive (2013/33/EU),^3^ where an entire chapter (IV) is dedicated to the “provisions for vulnerable persons”^4^ (see also Freedman, 2019; Pétin, 2016). Additionally, in the last few years, international humanitarian organizations, as well as national governments,^5^ have invested a considerable amount of resources in developing tools to individuate “vulnerable” people, especially in the context of refugees’ reception, such as the Vulnerability Assessment Framework (VAF)^6^ and the Vulnerability Screening Tool (VST)^7^ (Heidbrink, 2020; Sözer, 2020). Consequently, vulnerability has also become a technical and bureaucratic category aimed at directing assistance within humanitarian aid and refugee reception (Sözer, 2019; Turner, 2019).

The use of this term is so widespread, within and beyond the field of migration, that vulnerability has become a “buzzword”(Brown et al., 2017). Implemented in a variety of fields and for a variety of purposes, both in academic research and in policy frameworks, vulnerability is often treated as a self-explanatory condition or phenomenon (Brown et al., 2017; Cole, 2016; Flegar, 2018; Hruschka & Leboeuf, 2019; Turner, 2019). Indeed, in the context of migration, vulnerability is often used to categorize migrants in specific groups based on precise characteristics, without specifying how the notion is conceptually understood or defined (Sözer, 2019, 2020; Turner, 2019). However, we argue that vulnerability is neither conceptually straightforward nor as morally and politically neutral as its popularity may suggest.

A common and systematic understanding of the notion of vulnerability seems to be missing, with conceptualizations and definitions varying enormously not only across fields of research but at times also within them (Brown, 2011; Brown et al., 2017; Gorur, 2015; Hruschka & Leboeuf, 2019; Virokannas et al., 2020). Moreover, scholars across social sciences have highlighted the moral as well as political meaning intrinsic or ascribed to the concept of vulnerability and how this notion may become a tool for discrimination, control, or even oppression, when implemented in social policies (Brown, 2011; Casadei, 2018; Lind, 2019; Smith & Waite, 2019; Virokannas et al., 2020). Sözer (2019) argues that, in the context of refugee reception, the self-evident positive moral value attributed by humanitarianism to vulnerability renders the notion “immune from scrutiny” and “delays any discussion about what it entails” (Sözer, 2019, p. 2). Yet, vulnerability can be instrumentalized to foster particular political agendas, thus producing problematic humanitarian practices and discourses (Sözer, 2019; Turner, 2019). Given this conceptual and political complexity, Brown (2011) argues that instead of taking vulnerability at face value, the concept should be “handled with more care” and that “Academics in the social sciences need to […] start interrogating the conceptual boundaries of the notion” (Brown, 2011, p. 319).

The current paper aims to answer Brown’s call by providing a critical conceptual analysis of the notion of vulnerability in the context of migration. Specifically, this article has two consecutive goals. First, taking an interdisciplinary approach, we want to critically explore and highlight the conceptual complexity hidden behind this notion as well as the potential (un)intended consequences of its implementation in the context of migration. Secondly, we aim to show the potential analytical power of the concept in this field by proposing a new conceptual model for the understanding and study of vulnerability in relation to migration. To achieve these goals, the paper will first (1) provide a broad (albeit not comprehensive) overview of how vulnerability is commonly defined in different fields and in its relation to other popular concepts, namely risk, capacity, autonomy, and dependency. The second Sect. (2) will highlight how, implicitly or explicitly, different conceptualizations or types of vulnerability have been employed in previous literature and policy documents, mainly characterizing it as innate, situational, or structural. In the following Sect. (3), we will provide a detailed overview of the main critiques which have been moved against the concept of vulnerability and its implementation in social and migration policy. Drawing from this rich overview, the final Sect. (4) outlines our proposal for a new multi-level model, which embraces both the conceptual and the political complexity of vulnerability outlined in previous sections, providing a conceptual map of the levels of study and action, notions, and processes behind this concept.

## Defining Vulnerability

In order to elaborate a conceptualization of vulnerability within the context of migration, it is important to acknowledge the vast popularity of the term across different fields of study and the multiplicity of meaning that seems to hide behind it. As noted previously, a broad spectrum of definitions and understandings of the notion of vulnerability exists across the diverse academic fields that have engaged with the term. However, we can observe that, across disciplines, this notion has often been discussed in relation to other key concepts when attempting to define it. The current section will examine definitions of vulnerability, both within and outside the field of migration studies, in relation to the concepts of risk, capacity, autonomy, and dependency.

### Risk

A recurrent definition of vulnerability describes it as “an internal risk factor of the subject or a system that is exposed to a hazard and corresponds to its intrinsic tendency to be affected, or susceptible to damage” (Paul, 2014, p. 1). This same definition is employed (implicitly or explicitly) in various disciplines, only varying in terms of which type of “system” is considered (Gallopín, 2006; Paul, 2014; Singh et al., 2014). Thus, in disaster management and environmental science, the focus would be on ecological, natural, or biophysical systems; sociology would instead consider socio-political systems; for studies in economics, the system of reference may be the international financial market, household incomes, or national welfare systems; and health and medical studies would understand “system” as biological, thus focusing primarily on the human body (Alwang et al., 2001; Gallopín, 2006; Gilroy, 2006; Singh et al., 2014). The type of system considered would also determine the “content” of the risk examined. Following the systems mentioned above, this could mean respectively the risk of being affected by a natural disaster, the risk of not benefitting from the same rights as other citizens, the risk of financial loss and poverty, or the risk of contracting a disease. For an example from the medical field, it suffices to think of the current COVID-19 pandemic, in which some people or groups of people are considered vulnerable as they have a higher risk to develop life-threatening symptoms after contracting the virus. For the field of economics, an important example of the definition of vulnerability in terms of risks comes from the literature on poverty (Alwang et al., 2001; Gorur, 2015). Here, vulnerability is often defined as the level of likelihood (or risk) that a household may fall underneath the poverty line. Interestingly, as poverty is a dynamic process, with households moving below and above the poverty line in a certain period of time, vulnerability has become a useful analytical tool to help measure poverty and its determinants (Alwang et al., 2001).

Also with regard to migration, vulnerability is sometimes understood in terms of risk. For example, the Glossary on Migration of the International Organization for Migration (IOM, 2019) defines a *Vulnerable Group* as “Depending on the context, any group or sector of society […] that is at higher *risk* of being subjected to discriminatory practices, violence, social disadvantage, or economic hardship than other groups within the State. These groups are also at higher risk in periods of conflict, crisis or disasters” (italics added). Hence, according to this definition, being part of a vulnerable group means being at higher risk than members of other groups within a specific administrative and legislative entity: *the State*. However, the type of risk involved is not defined clearly but may involve a variety of hazards (i.e., discriminatory practices, violence, social disadvantage, or economic hardship).

When focusing specifically on migrants, the same glossary (IOM, 2019) adapts a definition provided by the United Nations High Commissioner for Human Rights (HCHR). It thus defines *Migrants in vulnerable situations* as “Migrants who are unable to effectively enjoy their human rights, are at increased *risk* of violations and abuse and who, accordingly, are entitled to call on a duty bearer’s heightened duty of care” (italics added). In this case, the content of the risk that characterizes the vulnerable situation in which migrants may find themselves is explicitly indicated as *violations or abuse of human rights.* This understanding of vulnerability seems in line with the findings of Peroni and Timmer (2013), who have analyzed how the European Court of Human Rights has been increasingly relying on the use of “vulnerable groups” in its judgments to ensure the safeguarding of human rights of marginalized groups, including migrants and particularly asylum seekers. Similarly, Hruschka and Leboeuf (2019) highlight how, according to the experts in a high-level-stakeholder meeting on migration governance, “the notion of ‘vulnerability’ acknowledges that those forced to flee and seek protection are exposed to higher risks of enduring human rights violations.” It is also interesting to note how, regardless of the specific or varied content of risk, both these definitions seem to trace back a state of vulnerability to *structural* conditions or situations rather than *individual* attributes.

### Capacity

Oftentimes, definitions of vulnerability also refer to the idea of capacity. For example, in socio-ecological systems, vulnerability is regularly referred to as the low capacity or inability of a system to respond to or withstand the perturbations of external stressors (Gallopín, 2006; Paul, 2014). Hence, a system is considered vulnerable when it possesses a low or null capacity of coping with hazards. Similarly, in research ethics, individuals or groups are commonly identified as vulnerable if they do not have the capacity “to safeguard their own rational interests” (Luna, 2009). As an example, Luna explains how scholars doing biomedical research with seriously ill people consider this group vulnerable, as they are increasingly susceptible to “enticing offers” of alleviating their condition, which reduces their capacity to safeguard their interests. In line with this reasoning, in a Guidance Note on *Research on refugees, asylum seekers & migrants* produced by the Directorate-General for Research and Innovation of the European Commission, researchers are worn to “not create unjustified expectations in participants” when interacting with “vulnerable groups” and “to avoid undue inducement”.^8^

Reference to capacity can also be found in the definition of *Vulnerability* elaborated by IOM (IOM, 2019): “Within a migration context, vulnerability is the *limited capacity* to avoid, resist, cope with, or recover from harm. This limited capacity is the result of the unique interaction of individual, household, community, and structural characteristics and conditions” (italics added). On a superficial read, this definition seems perfectly in line with the other glossary items reported above. However, we argue that the use of the concept of capacity rather than risk has important implications for our understanding of vulnerability and possible implementations of this concept in the context of migration. Firstly, defining vulnerability as “limited capacity” of an individual or group to “avoid, resist, cope with, or recover from harm” (IOM, 2019) implies a “deficiency” or at least a deviation from a norm where subjects are capable of performing such activities. This view, in line with a neo-liberal view of the political subject, can be problematic when applied to the concept of vulnerability as it could result in forms of stigmatization and marginalization of those people deemed vulnerable (Brown et al., 2017; Casadei, 2018; Fineman, 2008, 2010). Second, this definition brings important implications, as it shifts the focus from the sources of harm or vulnerability to the supposedly limited capacity of some individuals or groups to face them. The aim of the second part of IOM’s definition could then be interpreted as an attempt to counteract this “liberal shift,” pointing out that the “limited capacity” can not only be attributed to individual traits, but it is “the result of the unique interaction of individual, household, community, and structural characteristics and conditions.”

### Autonomy and Dependency

In line with the definition reported above, if vulnerability implies reduced (or absence of) capacity, it may also imply a diminished level of autonomy and thus higher dependency. It is then within the much broader critical debate on concepts of autonomy and dependency in the gender and feminist literature of the last decades that we find reference to (and revaluation of) the concept of vulnerability. Without going in depth in this extremely vast and varied corpus of literature, which applies a “feminist lens” to the discussion of these subjects in several fields (law, political science, bioethics, etc.), we may still draw interesting insights for the understanding of vulnerability.

The main argument behind these studies is that modern western societies are permeated by a masculinist ideology that sees the individual as completely autonomous, independent, and invulnerable (Brown et al., 2017; Cole, 2016; Fineman, 2010). The aspiration, or rather myth, of invulnerability becomes then the standard by which everybody is not only categorized but also judged. Those who are “capable” (or invulnerable) are those who can take their rightful place in society, and those who have limited capacity (vulnerable) cannot actively participate in society and must thus be protected (Peroni & Timmer, 2013). In this marginalized group of “vulnerables” and “dependents,” women have clearly been cast for centuries, together with racial/ethnic minorities and migrants.

From this feminist reflection on the concept of autonomy and dependency, a new corpus of literature has emerged that calls for a different understanding of vulnerability and a re-appropriation of the term for social change (Anderson, 2003; Brown et al., 2017; Butler, 2016; Cole, 2016; Fineman, 2008, 2010). That is, if we acknowledge the negative connotation attributed to vulnerability and dependency as the product of masculinist ideology, we can then challenge its epistemological origin. Indeed, several feminist scholars have argued that total independence and invulnerability are a myth, and thus unachievable not only by some groups of individuals but by any human being. It follows that our very embodied (as in possessing a physical body) and social (as in relational) nature makes any human being dependent and vulnerable to a certain extent.

Starting from this basic assumption, a new definition of vulnerability with more positive “moral” connotations can emerge. Vulnerability is not something to be avoided but a part of the human experience that, if acknowledged and owned, can bring about substantial change. From this “universalistic” definition of vulnerability stems the pioneering work of Martha Albertson Fineman proposing the introduction of a new “responsive state” governing “vulnerable bodies,” as an alternative to the liberal protection model based on identity politics (Fineman, 2008, 2010). In this complex and, in some ways, provocative proposal, the author claims that the universal understanding of vulnerability “must be at the heart of our concept of social and state responsibility” (Fineman, 2008, p. 8). Hence, “the ‘vulnerable subject’ must replace the autonomous and independent subject asserted in the liberal tradition,” as it is “far more representative of actual lived experience and the human condition” (Fineman, 2008, p. 2).

Similarly, Butler (2016) argues that the binary opposition between political agency and vulnerability, whereby a political subject is expected to “overcome” its vulnerability to enter the political arena, is the product of a masculinist ideal. The author continues by proposing “that vulnerability, understood as a deliberate exposure to power, is part of the very meaning of political resistance as an embodied enactment”(Butler, 2016). Therefore, the “mobilization of vulnerability” becomes the key to collective forms of political resistance and social change. Note that for Butler, vulnerability, although universal, should not be reduced to “injurability” nor to a subjective disposition, as its distribution is unequal in society. Although offering only a small insight into the extensive feminist literature on vulnerability, these two examples are meant to illustrate how vulnerability can and has been employed as a critical and political concept. Indeed, Fineman claims that “the concept of vulnerability can act as a heuristic device” (Fineman, 2008, p. 9) allowing us to uncover hidden and latent relationships and critically explore societal and political institutions (Fineman, 2010; Peroni & Timmer, 2013).

## Conceptualizing Vulnerability

Moving beyond (or beside) formal definitions of vulnerability, we now examine how the concept has been characterized and then employed in the literature, where different types of vulnerability seem to emerge. In the following section, we identify and describe three main conceptualizations of this notion, which characterize vulnerability respectively as the product of innate/natural characteristics (*innate vulnerability*); as the product of past, present, or future situations and experiences (s*ituational vulnerability*); or as the product of structural characteristics and dynamics (*structural vulnerability*). It is important to note that the focus on one or more of these types of vulnerability in the literature on migration is not always the result of a clear theoretical approach but rather appears at times to be implicit or even arbitrary. However, different characterizations of vulnerability lead to specific conceptual implications and policy trajectories (Brown, 2011). It becomes thus essential for any migration scholar trying to engage with this topic to recognize the different nuances of meaning given to this concept in previous literature and their theoretical and political implications.

### Innate Vulnerability

A first common understanding of vulnerability sees it as an *innate* or natural condition characterizing certain persons or groups (Brown et al., 2017). In the literature conceptualizing vulnerability in its connection with risk, the “innately” vulnerable are those who are in a state of permanent risk because of “natural” characteristics (Virokannas et al., 2020). Traditionally, studies employing this conceptualization have focused on gender,^9^ disability or chronic medical conditions, and age. Such understanding of “innate” vulnerability is often employed also in the migration context and especially in refugee reception to identify the “most vulnerable migrants.” Indeed, Flegar (2018) has shown how in its policy documents the UNHCR has often relied on this understanding of “innate” vulnerability, albeit implicitly, to describe women, children, elderly, and disabled people. It is also important to note that this conceptualization may have political consequences. That is, if vulnerability is understood as an innate characteristic of a person, measures and policies addressing it will need necessarily to be protective in nature, as the condition of vulnerability is by definition inherent and inevitable.

Moreover, conceiving vulnerability as innate presents some conceptual limitations, as the case of age as a factor in determining vulnerability well illustrates. It is commonly accepted that children or older people are “naturally” more vulnerable than other age groups. However, connecting vulnerability to certain stages of life implies a paradox: it is simultaneously “inevitable”—age cannot be chosen or modified—and “transient”; as time passes, the “natural” condition of vulnerability continuously changes (Heidbrink, 2020). The debate on coming-of-age Unaccompanied Minors (UAM) within the context of forced migration governance well exemplifies this paradoxical definition and its consequences in policy. A UAM is a person under the age of 18 who, upon entering a country, is not accompanied by the adult responsible for them by law or practice (European Migration Network (EMN), 2018). Especially in the case of a request for international protection, this group is commonly considered in migration policy and public discourse, among the “most vulnerable” and “deserving” of special protective measures (Flegar, 2018; Ní Raghallaigh & Thornton, 2017). However, when these young (and thus vulnerable) people turn eighteen and are legally considered adults, they suddenly lose their “legal vulnerable status.” This loss of vulnerable status does not only entail a loss of essential protections, but it may also cause and exacerbate new vulnerabilities such as limited access to services and healthcare, homelessness, and deportation (Heidbrink, 2020; Ní Raghallaigh & Thornton, 2017). Moreover, Heidbrink’s (2020) research on policies and practices of reception of UAM in Italy has highlighted how the transient nature of age has been strategically used to discharge the state’s responsibility toward young migrants: according to some of Heidbrink’s informants, the long waiting times of the system of reception are deliberately set toward the “aging-out” of UAM (Heidbrink, 2020).

### Situational Vulnerability

On the opposite side of the spectrum, a consistent corpus of literature has emphasized the situational character of vulnerability. In this type of accounts, certain people or groups are considered vulnerable because of a specific situation or experience that they have been through (e.g., victims of violence), are living through (e.g., homeless people), or may be exposed to (e.g., inhabitants of a seismic area). Contrary to the innate conceptualization of vulnerability, situational vulnerability seems to better account for the possibility of change through time as well as agency and would thus also move toward proactive and not only protective policies aimed at helping people “leave the situation of vulnerability.” Indeed, Luna (2009) highlights that conceiving vulnerability not as a “permanent and categorical condition […] that persists throughout its existence” but rather in relation to a specific situation that “makes or renders someone vulnerable” means also recognizing its *relational* nature. Relational vulnerability is then conceptualized as “the relation between a person or group of persons and the circumstances or the context” (Luna, 2009, p. 129). It follows that someone’s vulnerability results from such situational interaction between contextual circumstances and personal characteristics.

In the context of migration, this approach may see the migration process as a situational condition of vulnerability. For example, Flegar (2018) highlights how in the policy documents of IOM the “migration or displacement context itself is also referred to as generally contributing to vulnerability due to unfamiliarity with a new context, unsafe migration conditions, irregular status, a lack of social security entitlements and barriers to or generally weak protection” (Flegar, 2018, p. 380). Forced migrants as a group are often considered (implicitly or explicitly) a vulnerable group because of the situation in their country of origin that “forced” their migratory movement, which may include direct and indirect experiences of violence, persecution, famine, or other forms of peril (Sözer, 2020). Similarly, the European Court of Human Rights has recognized asylum seekers as a vulnerable group (in the *M.S.S. v. Belgium and Greece* judgment), but because of the situation of legal disadvantage they are experiencing in the present, in the country of destination (see ECRE, 2017). Alternatively, in a note on “Migrants in vulnerable situations,” the UNHCR specifically refers to the “Situational Vulnerability” of migrants as the “circumstances en-route or in countries of destination that render migrants at risk” (UNHCR, 2017).

Interestingly, the same note acknowledges also another “category” of vulnerability more in line with innate conceptualizations of vulnerability: the document identifies “individual vulnerability” as a situation of vulnerability that “relates to individual characteristics or circumstances which place a person at particular risk such as that experienced by: children, […] older people; those with […] disabilities;…” (UNHCR, 2017, p. 2). EU policy documents also seem to employ both conceptualizations of vulnerability, innate, and situational, when identifying “vulnerable migrants.” For instance, Article 21 of the Reception Conditions Directive (2013/33/EU)^10^ provides a list of who may be considered a “vulnerable person” (and thus should be awarded special provisions in the national laws), ranging from “minors, unaccompanied minors, disabled people, elderly people, …” (vulnerable because of *innate* characteristics) to “persons who have been subjected to torture, rape or other serious forms of psychological, physical or sexual violence …” (vulnerable because of experiences or *situations* of violence).

### Structural Vulnerability

Several authors have focused their attention on the structural grounds and dynamics that produce and reproduce vulnerability (Cole, 2016; Quesada et al., 2011; Szkupinski Quiroga et al., 2014; Virokannas et al., 2020). The basic assumption behind this conceptualization is that any person or group is positioned or situated in a specific context and is affected by this context (Brown, 2011; Casadei, 2018; Luna, 2009; Virokannas et al., 2020). Within the fields of human ecology and disaster management, the focus on the structure aims at reducing the risk or “pre-disposition” to (natural) hazards by identifying structural components that make a “system” (here understood as a group of people, community, city, but also a natural space or infrastructure) more vulnerable (Gorur, 2015; Paul, 2014). Within social sciences and legal studies, the focus on structural components of vulnerability is often a deliberate theoretical, critical, and perhaps also political, positioning against conceptualizations of vulnerability as a characteristic (both innate and situational) of a person or group described above. Indeed, Brown and colleagues warn that not only innate but also situational conceptualizations of vulnerability are quite normative and do not necessarily point to the complex system of social, institutional, or even ecological phenomena that have contributed to creating the situational condition of vulnerability (Brown et al., 2017). In other words, relating vulnerability to a set of personal or situational characteristics may run the risk of ignoring the social, institutional, legal, and economic conditions that create inequality, precarity, exploitation, and discrimination in society and thus also vulnerability in itself. As Luna points out, it is not her “natural” gender that may make a woman vulnerable, but it is rather the unequal misogynistic system in which she lives and which puts her in a subordinate position in comparison to men that creates the conditions for her vulnerability (Luna, 2009). Interestingly, Cole warns that also redefining vulnerability as a universal or ontological condition of any human being, as theorized by some feminist authors (see the “Autonomy and Dependency” section), may risk ignoring “the structural differentiation in who suffers which vulnerabilities” (Cole, 2016, p. 266). The author proceeds by claiming that what had started as a conceptual shift to better address forms of inequalities and injustice toward specific groups marginalized and stigmatized in society as “vulnerable” “could in fact prevent us from clearly perceiving what differentiates our vulnerability from the vulnerability of others” (Cole, 2016, p. 265).

The focus on structural vulnerability has two main implications. First, if vulnerability is structural, it is also socially, politically, geographically, and culturally situated. In this sense, vulnerability also becomes contextual and social, as it recognizes our inevitable interdependence as social beings to others as well as to the context of relations and structures within which we may negotiate and realize our autonomy and claim recognition (Casadei, 2018; Monno & Serreli, 2020). Secondly, the object of policies addressing and contrasting structural vulnerability should not be (solely) the single individual or group in a condition of vulnerability but the system that produced it. Vulnerability becomes then a valuable political tool to uncover and face systems of inequality and subjugation in society.

In regard to migration, Flegar (2018) has shown that IOM, within its policy documents especially in disaster contexts, “seems to suggest that […] the relationship between migration and vulnerability is relevant in three different ways: migration can cause vulnerability, migration can be a result of vulnerability and vulnerability can be a cause of non-migration” (Flegar, 2018, p. 380). While the first way seems in line with the conceptualization of situational vulnerability explored above, the latter two point to a conceptualization of vulnerability as structural. A straightforward example of how migration can be the *result* of structural vulnerability is the case of those refugees fleeing persecution: the structural social, political, and cultural characteristics of the country in which they live promote their vulnerability as members of a persecuted minority group, which results in their migratory movement. By contrast, structural vulnerability can also be the *cause of non-migration*: for example, when poor and marginalized groups are, because of a lack of resources (structurally determined), unable to move in the wake of natural disasters such as drought, flood, or famine.

Conceiving vulnerability as structural appears relevant not only when we understand migration in terms of mobility but also when we focus on the life of migrants within the country of arrival. Indeed, migrant communities may live in conditions of structural vulnerability because they are disproportionally exposed to forms of *structural violence*, which is often racialized (Quesada et al., 2011; Szkupinski Quiroga et al., 2014). Structural violence points to the systems of inequalities, exploitation, and oppression embedded in political and economic institutions, which promote unequal distributions of material resources and power across society (Farmer, 2004; Szkupinski Quiroga et al., 2014, p. 1725). Accordingly, the position of a person or group, such as migrants or ethnic minorities, within this social hierarchy of power dynamics and its legal and political effects determine their exposure to structural violence and thus their structural vulnerability (Quesada et al., 2011), which, at the individual level, “may manifest as psychic distress, depreciated subjectivities, social exclusion, and hierarchies of worthiness” (Szkupinski Quiroga et al., 2014, p. 1725). Following the same conceptual line, Heidbrink (2020) argues that the rise of far-right parties in Europe has encouraged foreign and domestic policies, such as the deal with the Libyan Coast guard stroked by the Italian government and backed by the EU, which “make migrants structurally vulnerable in countries of origin, transit, and arrival” (Heidbrink, 2020, p. 14). Thus, in the context of reception practices of an increasingly “hostile” Europe, “A disparate focus on individualized vulnerability shifts public perception away from the policies that render migrants vulnerable” (Heidbrink, 2020, p. 14).

## Critiquing Vulnerability

A considerable number of scholars have moved several critiques to the notion of vulnerability and its implementation in social and migration policy (Brown, 2011; Brown et al., 2017; Casadei, 2018; Sözer, 2020; Turner, 2019; Virokannas et al., 2020). The following section explores the main ones, shedding light on the implications or (un)intended consequences the concept of vulnerability may bring in its instrumentalization in politics, policy, and legal frameworks relating to migration as well as in its implementation as a conceptual tool in research on migration.

### Discriminating and Stigmatizing

Several authors have underlined how categorizing a group or individual as vulnerable can be *discriminating* and *stigmatizing* (Brown et al., 2017; Smith & Waite, 2019; Turner, 2019; Virokannas et al., 2020). We argue that this negative effect of implementing the notion of vulnerability may have two main causes. First, the stigmatizing and discriminatory effect of vulnerability can be the product of normative understandings of vulnerability. Indeed, using predefined categories of vulnerability often means reducing the complexity of the social, structural, and temporal dynamics that help create and shape the condition of vulnerability to one single characteristic of that person (Brown et al., 2017). This appears especially true when applying an *innate* conceptualization of vulnerability, which by definition cannot be changed and thus relegates a group or individual to an inevitable condition of vulnerability, increasing the risk of stigmatization. In the context of refugee reception, the increasing focus on the vulnerability of certain predefined groups of forced migrants has been noted to diminish the complexity of individuals’ identity, reducing them to that single characteristic that qualifies them as vulnerable (Smith & Waite, 2019). For example, a gendered conceptualization of vulnerability may lead to the stigmatization of refugee women as powerless and vulnerable victims (Freedman, 2019) but also of refugee men as non-vulnerable. As Turner explains (2019), the vulnerabilities experienced by migrant men have often been overlooked in this gendered framework, contributing to a discriminatory public discourse that often portrays refugee men as threatening or perpetrators.

Second, the discriminatory power of vulnerability can arguably be considered the result of an implicit moral judgment that sees the “vulnerable” as less capable, less autonomous, less rational, less competent, etc. In this sense, the stigmatizing effect of vulnerability is the product of liberal and masculinist ideals, discussed above, that see the “normal” subject as an “invulnerable,” independent, autonomous, and capable individual and, implicitly or explicitly, stigmatize those who do not conform to such ideal (Butler, 2016; Cole, 2016; Ferrarese, 2016; Fineman, 2008). As we mentioned previously, the term vulnerability hides important ethical and moral connotations that make its use challenging and often problematic (Brown, 2011; Cole, 2016; Fineman, 2010; Virokannas et al., 2020). It follows that vulnerability can become another powerful tool for not only marginalizing certain groups, such as migrants, at the peripheries of society in a process of “othering” but also subjugating them in a system of moral hierarchies (Grove & Zwi, 2006).

### Patronizing, Paternalistic, and Disempowering

On the same critical line, conceptualizations of vulnerability based on a neo-liberal self-deterministic perspective can also result in *paternalistic* and *patronizing* attitudes toward “vulnerable groups or people” (Brown, 2011; Brown et al., 2017; Fineman, 2008; Virokannas et al., 2020). If the “vulnerable” are “less then,” and their condition is relatively stable or permanent, then we, the “invulnerable,” “normal,” “capable” society and government have a responsibility to protect these groups, as they are not capable of doing so themselves. These implicit paternalistic and patronizing undertones characterize legal frameworks of protection and care, which have been often denounced as *disempowering* toward the members of the groups they “protect” and care for. Following this rhetoric and conceptualization of who is vulnerable (and why) and thus in need of care or protection, these systems often fail to recognize the agency and capabilities individuals in vulnerable conditions still possess and appear to pose powerlessness as a condition for access to protection (Brown et al., 2017; Butler, 2016; Heidbrink, 2020; Sözer, 2020; Turner, 2019). As Tew rhetorically asks, “Are there opportunities for them to challenge or renegotiate the extent of their need for protection?” (Tew quoted in: Clark, 2007, p.293). In the context of migration, this type of dynamic is clearly exemplified by asylum procedures and humanitarian aid, which promote an increasingly narrow definition of who *deserves* protection (Smith & Waite, 2019). Hence, in order to conform to such an idea of vulnerability, refugees are expected to present themselves (or perform) as incapable, powerless, and dependent on the “humanitarian generosity” of the powerful European states (Clark, 2007; Heidbrink, 2020; Sözer, 2020; Turner, 2019). As Heidbrink points out in the case of UAM, young refugees must not only prove their fulfillment of specific vulnerability criteria (in this case, their age as legal minors) but also “perform their vulnerability to qualify (or be assessed to qualify) for commensurate services and rights” (Heidbrink, 2020, p. 8). Interestingly, some authors have noticed how some asylum seekers enact strategic “performances” of powerlessness and vulnerabilities in order to fit into the asymmetric humanitarian rhetoric of protection just described (Clark, 2007; Turner, 2019). Paradoxically, these “strategic uses of vulnerability,” as described by Freedman (2019) in relation to women refugees, can be viewed as forms of agency or even acts of resistance against a disempowering system.

### Fostering Social Control and/or Oppression

When implemented in social policies, “under the guise of assistance and protection” (Brown et al., 2017, p. 501), vulnerability can also result in forms of social control and/or oppression (Brown, 2011; Casadei, 2018; Heidbrink, 2020; Virokannas et al., 2020). This can be enacted directly through policy interventions and policing activities, resulting in further marginalization of those deemed vulnerable (Brown, 2017), as well as indirectly, through the (voluntary) ignorance of structural systems producing inequalities and conditions of vulnerability, which will lead to their (re)production and, ultimately, to their justification (Casadei, 2018; Heidbrink, 2020). For example, in critical scholarship on neo-liberal humanitarianism, it has even been suggested that humanitarian work is an authoritarian effort, which reproduces power inequalities, promoting the control of the *powerless* beneficiaries by the *powerful* benefactors (Turner, 2019). Vulnerability is then seen as a pivotal instrument to enact this system of asymmetrical power relationships and control, as it promotes a narrative of marginalized groups “adapting to, rather than resisting, the circumstances they find themselves in” and thus contributes “to creat[ing] depoliticized (refugee) subjects” (Turner, 2019, p. 6). In other words, narratives of care, protection, and hospitality imply complete acceptance of humanitarian aid (in any form) as refugees' only possible answer, actively silencing them and thus promoting their control (Grove & Zwi, 2006). Indeed, as Butler suggests, “Once groups are marked as ‘vulnerable’ within human rights discourse or legal regimes, those groups become reified as definitionally ‘vulnerable’, fixed in a political position of powerlessness and lack of agency” (Butler, 2016, p. 15). Following this line of thought, according to Turner (2019), one of the current political struggles of refugees is to “resist the logic of ‘vulnerability’” applied in the humanitarian framework, which relegates them in “passive subject positions” and reduces them to “objects of interventions” (Turner, 2019, p. 16).

Laws and policies within deportation regimes and asylum systems of the increasingly hostile environments adopted by most (if not all) European states not only promote depoliticized and passive refugees but have also been noted to actively produce conditions of vulnerability and thus oppression (Heidbrink, 2020; Lind, 2019; Smith & Waite, 2019). The separation of the support provided to asylum seekers from the mainstream welfare provisions in the UK (Smith & Waite, 2019), the loss of benefits and protection of coming of age UAM (Heidbrink, 2020), and the state of deportability of undocumented migrants (De Genova, 2002) are all examples of how the state and its laws can actively “vulnerabilize” individuals. Lind (2019) goes one step further, proposing the use of “vulnerabilization” to describe “the political processes of creating the conditions for, defining and attributing vulnerability, which enables the governing of the ‘vulnerabilised’” (Lind, 2019, p. 341). Examining the case of undocumented children living in Sweden, Lind shows examples of how the state has created or heightened conditions of vulnerability (e.g., by denying asylum to a family and then excluding them from the welfare system once they enter irregularity), attributed vulnerability (e.g., by encouraging narratives that blame the undocumented parents for the vulnerability of their children), and through these means governed this group (e.g., by heightening conditions of vulnerability through hostile policies to the point that migrants are forced to “self-return” to their country of origin) (Lind, 2019).

### Exclusionary

As we have shown, critiques of vulnerability claim that conceiving it as a category or label attributed to certain individuals or groups may lead to discriminatory, stigmatizing, disempowering, and even controlling practices. Additionally, within the context of refugees’ reception and humanitarian aid, several authors have highlighted how the current commitment to identify and “protect” the “most vulnerable” refugees may also result in exclusionary practices and ultimately in narrowing protection of asylum seekers and further restrict access to services (Heidbrink, 2020; Hruschka & Leboeuf, 2019; Smith & Waite, 2019; Sözer, 2019, 2020; Turner, 2019). In recent years, the international community and humanitarian organizations have extensively focused on elaborating and deploying the notion of vulnerability by constructing tools to identify and measure it (such as the VAF and VST)^11^ and programs to help those refugees recognized as “vulnerable” (such as the ESSN – EU-Turkey Emergency Social Safety Network). The objective is to target and prioritize the protection and aid of those who are more in need of it, “the most vulnerable.” However, this more focused direction of humanitarian resources has recently come under some scrutiny as it carries significant implications.

First and foremost, it has been argued that focusing on the vulnerability of certain people, or certain vulnerable groups, among forced migrants, may reduce humanitarian assistance toward the entire group and limit access to protection and the rights established by international laws and conventions of those refugees deemed “less vulnerable” (Heidbrink, 2020; Hruschka & Leboeuf, 2019; Smith & Waite, 2019; Sözer, 2020). As Hruschka and Leboeuf (2019) caution, there is a “hidden exclusionary effect of the term vulnerability,” because using it in the policy discourse implies choosing “which vulnerabilities” to favor over others. Heidbrink (2020) brought a concrete example of how the notion of vulnerability has been employed to regulate the distribution of a form of aid within a group protected by international law, examining the emblematic event that occurred in 2018 in Italy: the so-called Diciotti standoff. When 177 migrants rescued in the Mediterranean by the Diciotti vessel of the Italian Coast Guard were not allowed to disembark at the port of Catania by the Italian government, Italian civil society and humanitarian actors “began to negotiate their release enlisting a *hierarchy of vulnerability*”(Heidbrink, 2020, p. 5). That is, based on criteria of vulnerability such as health conditions, age, and previous trauma, some migrants (“the most vulnerable”) were gradually allowed to leave the ship and get the aid they were entitled to, while the other 140 migrants had to wait on board the end of the 10 days standoff (Heidbrink, 2020). Naturally, no one disputes the need to disembark those migrants on the Diciotti who were sick as soon as possible. However, as Hruschka and Leboeuf point out (2019), it is also important to recognize that in the context of migration governance, “‘vulnerability’ is a double-edged sword: It acknowledges that persons with special needs are exposed to specific risks, but it may also be used as a tool to limit State protection and to undermine the existing protection framework” (Hruschka & Leboeuf, 2019, p. 3). Additionally, as Sözer noted, the deployment of the notion of vulnerability to direct humanitarian aid also highlights a moral shift in contemporary neo-liberal humanitarianism, whereby “it has become thinkable, morally permissible, reasonable, and even desirable to assist *only* to a fraction of the same forced migrant community” (Sözer, 2020, p. 2). Thus, an uneven distribution of humanitarian assistance is established, targeting the “‘right subjects of humanitarian empathy’ within forced migrants” (Sözer, 2020, p. 15).

Finally, another drawback of the use of vulnerability in targeting beneficiaries of international aid is the use of normative abstract categories, established by western humanitarian workers, in structured screening tools to identify “the most vulnerable.” Indeed, several authors have brought examples of “vulnerable people” that have been excluded by such fixed categories and have criticized them as incapable of capturing the complex structural (political, economical, and social) individual (biographical, psychological) and, we argue, also situational factors that play a role in the migration and integration processes and may contribute to create conditions of vulnerability (Crawley & Skleparis, 2018; Heidbrink, 2020; Sözer, 2020; Turner, 2019).

### Reifying (Vulnerability as a Label)

Also on a conceptual level, some authors have argued against the reifying effect of the vulnerability label. That is, labeling certain groups as vulnerable may run the risk to ignore differences within the group and thus ignoring who is actually living in a condition of vulnerability and who may not (or not as much) (Casadei, 2018; Clark, 2007; Heidbrink, 2020; Luna, 2009; Sözer, 2020). As Luna warns, “understanding vulnerability as a mere label does not help us to acknowledge, identify, and evaluate the life experiences of those deemed vulnerable” (Luna, 2009, p. 131). Additionally, ignoring different vulnerabilities within one group labeled as vulnerable also implies ignoring intersectionality or the fact that an individual may experience, in the words of Luna (2009), several *layers of vulnerability*. Following the example given by the author, a woman is not vulnerable as such but becomes so if she lives in a country that limits women’s reproductive rights. If this woman is also poor and illiterate, she will be arguably more vulnerable than a wealthy, educated woman living in the same country (Luna, 2009). Although it may appear straightforward, this approach implies a significant conceptual shift. Luna’s argument, as shared by other authors such as Clark (2007) and Heidbrink (2020), is to understand the concept of vulnerability dynamically, contextually, and relationally.

## Vulnerability as an Analytical Concept for the Study of Migration: a New Conceptual Model

The overview in the previous sections started from the critical standpoint that, perhaps because of its increasing popularity, vulnerability has often been mistakenly treated as a self-explanatory or self-evident condition. Thus far, the paper has been devoted to examining and highlighting the complexity hiding behind this now popular term, first by showing the multiplicity of understandings and conceptualizations of the notion and second underlining some conceptual, analytical, political, and moral consequences of their implementation. In the face of such complexity, what can we conclude about the analytical power of the concept of vulnerability in research on migration? Is the notion of vulnerability just too conceptually complex and too morally and politically ambivalent to be considered useful?

On the contrary, it is our contention that in the context of migration research, vulnerability can be a very powerful analytical concept, helping us better understand complex processes in migration and integration at different levels of analysis. That is not despite the complexity hidden behind the notion but precisely because of it. Our argument is that vulnerability in the context of migration should be understood as a multi-layered, dynamic, and embedded concept. Experiences of vulnerability cannot be captured by a fixed, measurable condition or even by a list of conditions that persist through time and space, nor can they be described as only *innate*, *situational*, or *structural*. Individuals and groups are situated in broader systems of socio-political hierarchies and power dynamics, and such structures are then reproduced in local systems and interpersonal relationships in people’s everyday lives, as well as reinterpreted and negotiated by individuals. Therefore, simultaneously or at different points in the life course, a person may be in a condition of vulnerability related to an innate characteristic, related to a specific situation, and/or related to structural conditions.

Indeed, we argue that to understand vulnerability, it is fundamental to account for temporality, both in terms of historical outlook into the geopolitical context, in (and across) which migrants operate over their lifetime, and in terms of personal development of single individuals, since, depending on the point in their lifespan, they might be differently affected by adverse events (Elder, 1998; Titzmann & Lee, 2018). Moreover, we propose that accounting for temporality can help to reconcile different definitions of vulnerability emerged from the overview. Looking at the future, it is reasonable to conceive vulnerability as the risk of a potentially negative event to affect individuals (see the “Risk” section). Looking at the present, vulnerability can be understood as the result of contingent conditions, which may be structurally determined, that limit the individual resources (or capacity) necessary to cope with negative events (see the “Capacity” section). Finally, examining individuals’ overall life course would point to more universal understandings of vulnerability, suggesting that at some point in life and to a certain extent, any individual has experienced vulnerability (as well as higher dependency and lower autonomy) (see the “Autonomy and Dependency” section). Thus, we argue that vulnerability can take different forms depending on the chosen temporal outlook. Regardless, each individual experience of vulnerability is always situated in a specific context, time, and developmental phase, and it is the product of interrelating structural, situational, social, biographical, and psychological characteristics.

**Fig. 1:**
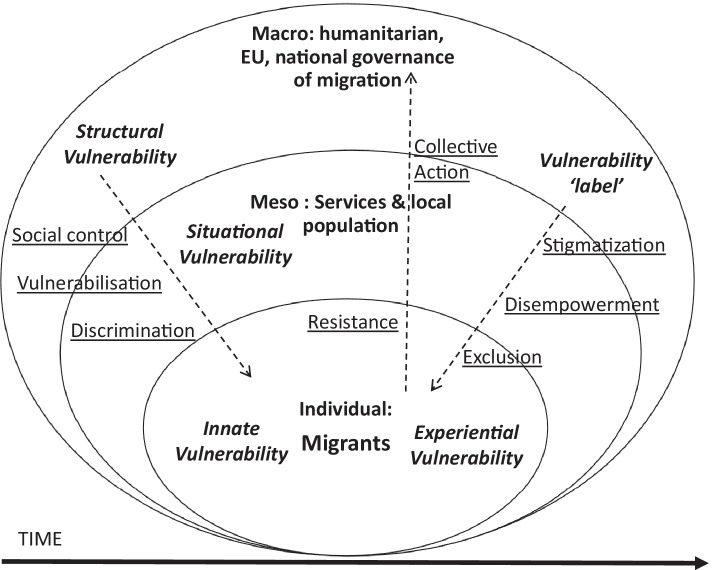
Conceptual model of vulnerability in the context of migration

Based on these considerations, we propose a new conceptual model of vulnerability (see Fig. 1) that wishes to systematize, at different levels of analysis, rather than discard the multiple conceptualizations described.^12^ Simultaneously, the model acknowledges the possible negative consequences of this notion’s implementation by including cross-level processes pointing at the interrelationships between levels of analysis.

At the macro level of the model, we see how the analysis of international and national legal frameworks of migration governance and humanitarian aid as well as the national systems of opportunity structures can point to migrants’ *structural vulnerability*, as described above (see the “Structural Vulnerability” section). Indeed, structural vulnerability can become a useful tool, or in Fineman’s words, a heuristic device (2010), to individuate and expose unequal distributions of power and resources embedded in political and economic organizations, which disproportionally affect migrant communities (Quesada et al., 2011; Szkupinski Quiroga et al., 2014). Structural inequalities are reproduced in local services and interpersonal relationships (meso level) and affect individual migrants through processes of discrimination (see the “Discriminating and Stigmatizing” section). Investigating vulnerability at this level also allows us to examine how systems of migration and asylum laws can contribute directly to creating conditions of vulnerability or, in other words, can actively “vulnerabilize” migrant individuals (Heidbrink, 2020; Lind, 2019) (see the “Fostering Social Control and/or Oppression” section). We then identify “vulnerabilization” as another cross-level process where macro structures, in this case legal systems, are implemented in local institutions (such as police stations, hospitals and health services, and employment agencies) and then directly affect the everyday lives of migrant groups and individuals. Similarly, systems of inequality and migration governance can also shape migrants’ experiences and positions in society by promoting processes of social control (see the “Fostering Social Control and/or Oppression” section).

Moreover, as this notion becomes more embedded into migration policies and laws, legal and political systems can not only create or promote conditions of vulnerability but also contribute to formalizing normative definitions of vulnerability in what we would call the *bureaucratic vulnerability label*, used to identify the “vulnerable” migrants. In this context, we do not dispute that the implementation of vulnerability may contribute to highlighting the systems of inequality discussed before and, more importantly, may promote assistance to those individuals and groups who are more affected by them. However, as we have shown above, the systematization of individuals’ experiences and conditions of vulnerability in predefined categories can give origin to processes that negatively impact those individuals by stigmatizing them (see the “Discriminating and Stigmatizing” section), disempowering them (see the “Patronizing, Paternalistic, and Disempowering” section), and even excluding some of them from the aid they should be entitled to (see the “Exclusionary” section). We propose that all these negative consequences can also be conceived as cross-level processes that originate at the macro level with the creation of the *bureaucratic vulnerability label* and directly affect the everyday lives and experiences of vulnerability of migrant individuals.

Structural and bureaucratic vulnerability, as well as the processes just highlighted, are then (re)produced at the meso level. This level includes the complex systems of local institutions and services interacting with migrant communities as well as the networks of interpersonal relationships between migrants and non-migrant residents embedded in a specific space and time. It is here that the interaction between contextual circumstances and personal characteristics, experiences, and aspirations of individual migrants occurs, resulting in forms of *situational vulnerability* (see the “Situational Vulnerability” section). As the area of negotiation between individuals and institutions, the meso level is also where the “performances” or “strategic uses of vulnerability” to gain access to systems of services or protection, highlighted above (see the “Patronizing, Paternalistic, and Disempowering” section), can occur. Interestingly, Mesarič and Vacchelli (2019) show how also third-sector organizations strategically invoke normative and essentialized understandings of vulnerability in order to legitimize their services and thus secure funding from governmental institutions.

Finally, at the individual level, we argue that, although it is indisputable that individuals with certain (innate) characteristics or positions in society face more challenges than others, pointing to their *innate vulnerability* (see the “Innate Vulnerability” section), the extent to which a single individual experiences vulnerability cannot be predetermined on the basis of fixed, innate characteristics. Paraphrasing Gamsakhurdia (2019), we argue that beyond common characteristics and events, there are objective and subjective factors which ensure that vulnerability (as he argued for proculturation) is always a uniquely personal experience. Thus, at this level, we propose to consider an additional form of vulnerability, which we would call *experiential vulnerability*. Inspired by critiques to bi-dimensional models of acculturation (Gamsakhurdia, 2019), this notion points to the uniqueness of each migrant’s life experiences, thus also acknowledging inter-individual variance within a vulnerable group as well as possible instances of intersectionality. Additionally, as Gamsakhurdia (2018, p. 549) points out: “We should consider [the] possibility that different persons may react differently to the same conditions due to their distinctive interpretations of those conditions.” Therefore, the notion of *experiential vulnerability* aims also to capture how each individual migrant emotionally and psychologically processes such “vulnerable experiences” within their life course or, in other words, how they subjectively construct their vulnerability, highlighting both processes of adaptation and the agency of each migrant facing conditions of vulnerability.

Indeed, following Butler (2016), we maintain that vulnerability does not necessarily imply a lack of agency or powerlessness but that, on the contrary, it could actively enable agency through forms of resistance. Furthermore, we suggest that when acts of individual everyday resistance are shared and organized through dialogical networks, vulnerability could be mobilized to give life to cross-level processes of political resistance and collective action. Through these processes, migrant communities could re-shape the local systems of services and institutions and inter-group relationships characterizing their lived context and even bring more structural social changes in national and international socio-political systems.

## Conclusion

The concept of vulnerability occupies a prominent role in both academic literature on migration and migration policy. Although its adoption in humanitarian and protection frameworks has recently come under some scrutiny by scholars in the field, the complex and multifaceted nature of vulnerability is often overlooked.^13^ Its widespread use has led many scholars to treat the concept as a self-explanatory phenomenon without problematizing neither its conceptualization nor its use and its possible negative consequences.

However, in this paper, we have shown how the concept of vulnerability is as complex as it is powerful. First, we have unpacked the plurality and complexity of meaning hiding behind this notion by outlining its relation with other key concepts. Second, focusing on how vulnerability has been characterized or conceptualized in previous literature and policy frameworks, we have outlined three main “types” of vulnerability and discussed their conceptual implications. Third, providing an overview of the main critiques against vulnerability, we explored the possible negative consequences that may arise from its use both in policy and in academic work and showed how vulnerability could result in forms of discrimination, stigmatization, exclusion, disempowering, and paternalistic practices and even aid social control and oppression.

Finally, in the last section, we have outlined our proposal for a new conceptual model for understanding vulnerability in the context of migration. By embracing the multi-layered conceptual complexity of this notion and acknowledging its embedded and dynamic nature, it was shown how vulnerability can become a useful analytical lens to examine experiences, processes, dynamics, and systems related to migratory movements and settlement.

To conclude, it is necessary to situate individuals deemed “vulnerable”, or “not vulnerable enough”, in the cultural, geographical, political, and temporal system in which they operate and develop, and critically evaluate the legal, moral, and political implications of the vulnerability label, to ensure that migrants will not further be stigmatized, controlled, and marginalized as an individual or group. Vulnerability can uncover systems of discrimination and oppression affecting migrant communities while simultaneously acknowledging the agentic power of migrant individuals and their unique experiences. This way, in opposition to disempowering narratives of vulnerability often adopted in discourses and policies around migration, vulnerability has the potential to promote migrants’ empowerment and social change.

## Footnotes

1 DIRECTIVE 2011/95/EU OF THE EUROPEAN PARLIAMENT AND OF THE COUNCIL of 13 December 2011 on standards for the qualification of third-country nationals or stateless persons as beneficiaries of international protection, for a uniform status for refugees or for persons eligible for subsidiary protection, and for the content of the protection granted (recast).

2 DIRECTIVE 2013/32/EU OF THE EUROPEAN PARLIAMENT AND OF THE COUNCIL of 26 June 2013 on common procedures for granting and withdrawing international protection (recast).

3 DIRECTIVE 2013/33/EU OF THE EUROPEAN PARLIAMENT AND OF THE COUNCIL of 26 June 2013 laying down standards for the reception of applicants for international protection (recast).

4 For a comprehensive analysis of the legal concept of vulnerability, see IARMJ-Europe under contract to EASO. (2021). *Judicial analysis Vulnerability in the context of applications for international protection*. EASO Professional Development Series for members of courts and tribunals. https://doi.org/10.2847/2759

5 For an overview of the policy and practices across EU Member States, see EASO (2020). *EASO Asylum Report 2020: Annual Report on the Situation of Asylum in the European Union*. https://doi.org/10.2847/531878

6 Developed by UNHCR in 2013 among Syrian refugees in Jordan.

7 Developed by UNHCR and the International Detention Coalition in 2016.

8 European Commission (2020). Guidance note — Research on refugees, asylum seekers and migrants: V1.1. https://ec.europa.eu/research/participants/data/ref/h2020/other/hi/guide_research-refugees-migrants_en.pdf

9 See Virokannas et al. (2020), p.331–333, for a short review of social science literature relating vulnerability and gender.

10 DIRECTIVE 2013/33/EU OF THE EUROPEAN PARLIAMENT AND OF THE COUNCIL of 26 June 2013 laying down standards for the reception of applicants for international protection (recast).

11 See Introduction

12 The proposed model was partially inspired by the heuristic model of Liquid Integration developed in the context of the MIMY project by fellow consortium members. See Skrobanek, Jan, in collaboration with Jobst, Solvejg, Grabowska, Izabela and Ryan, Louise (2020). “Liquid integration” of vulnerable migrant youth. Some general considerations. *Bergen Studies in Sociology Series*, (15/2021). https://bora.uib.no/bora-xmlui/bitstream/handle/11250/2830320/Liquid_integration_regarding_vulnerable_migrant_youth_final.pdf?sequence=1

13 For a comprehensive and in-depth analysis of the implementation and translation of vulnerability into legal protection regimes, we invite you to refer to the ongoing project funded by the EU, VULNER—Vulnerabilities Under the Global Protection Regime: https://www.vulner.eu/3169/about
